# Photonic crystal nanocavity assisted rejection ratio tunable notch microwave photonic filter

**DOI:** 10.1038/srep40223

**Published:** 2017-01-09

**Authors:** Yun Long, Jinsong Xia, Yong Zhang, Jianji Dong, Jian Wang

**Affiliations:** 1 Wuhan National Laboratory for Optoelectronics, School of Optical and Electronic Information, Huazhong University of Science and Technology, Wuhan 430074, Hubei, China

## Abstract

Driven by the increasing demand on handing microwave signals with compact device, low power consumption, high efficiency and high reliability, it is highly desired to generate, distribute, and process microwave signals using photonic integrated circuits. Silicon photonics offers a promising platform facilitating ultracompact microwave photonic signal processing assisted by silicon nanophotonic devices. In this paper, we propose, theoretically analyze and experimentally demonstrate a simple scheme to realize ultracompact rejection ratio tunable notch microwave photonic filter (MPF) based on a silicon photonic crystal (PhC) nanocavity with fixed extinction ratio. Using a conventional modulation scheme with only a single phase modulator (PM), the rejection ratio of the presented MPF can be tuned from about 10 dB to beyond 60 dB. Moreover, the central frequency tunable operation in the high rejection ratio region is also demonstrated in the experiment.

Silicon photonics has become one of the most promising photonic integration platforms owing to its small footprint, reduced power consumption, and availability of complementary metal-oxide-semiconductor (CMOS) fabrication technology^1^. Because of its unprecedented small size for potential large scale integration, silicon photonic crystal (PhC) nanocavity is of great importance to accelerate the success of silicon photonics^2^,^3^,^4^,^5^,^6^. In traditional scenario, large scale photonic integration is usually driven by the digital applications, such as high capacity optical communications and optical interconnects technologies. In recent years, due to the strong requirements in handling analog signals with low power, high efficient and high reliability, the use of integrated photonics technology to generate, distribute, process and analyze microwave signals has also attracted more and more research interests^7^,^8^,^9^.

Microwave photonic filter (MPF), which can be employed to process microwave signals in the optical domain by using photonic devices, is a key element in microwave photonic systems. Several approaches to realizing MPFs have been proposed and demonstrated based on bulky fiber based devices^10^,^11^,^12^,^13^. These configurations are relatively expensive, power consuming, and deficient in flexibility and stability. Compared to these fiber devices, silicon-on-insulator (SOI) based waveguides can offer distinct advantages of increased stability and reliability, low cost, small footprints, and compatibility with other integrated optoelectronic devices. Recently, some MPFs based on SOI microring resonator, microdisk resonator, and Mach–Zehnder interferometer have been proposed and demonstrated showing superior characteristics^14^,^15^,^16^,^17^,^18^.

Tunability of MPFs is highly desirable in practical applications to facilitate flexible performance. Previously reported tunable or reconfigurable MPFs mainly focus on tuning the bandwidth and central frequency^10^,^13^,^19^,^20^,^21^. Besides tuning of bandwidth and central frequency, the stop-band rejection ratio tunability is also highly desirable for a bandstop microwave filter. Specifically, in cognitive systems, the rejection ratio of a microwave filter need to be tunable for handing different needs of interference rejection^22^,^23^. In electrical domain, when constructing a microwave filter with continuous rejection ratio tunability, resonators with tunable coupling to a transmission line are usually utilized^22^,^23^,^24^,^25^,^26^,^27^. The operation frequency of these filters are limited to a few GHz. To increase the operation frequency, a MPF have been proposed to construct rejection ratio tunable microwave filters in optical domain, showing superior performance^28^. It is based on a Sagnac loop assisted by a polarization beam splitter and a linearly chirped fiber Bragg grating. The rejection ratio of the MPF can be tuned from 0 to 37 dB. A central frequency tuning range from 2.5 to 13 GHz is also observed. However, the configuration of this rejection ratio tunable MPF is relatively complicated, and the obtained maximum rejection ratio is not very large owing to the limited maximum split ratio of the polarization beam splitter. Moreover, the application of this MPF is also limited by its periodic response which results from large loop path difference between the two taps. So far, achieving a rejection ratio tunable single stop-band notch MPF with wide rejection ratio and central frequency tuning ranges is still challenging.

In this paper, we propose a simple scheme to realize rejection ratio tunable single stop-band notch MPF with wide tuning range based on a silicon PhC nanocavity with fixed extinction ratio. We use the combination of a conventional phase modulator (PM), a tunable bandpass filter (TBF), and a silicon PhC nanocavity to manipulate the phase and amplitude of optical sidebands. Lower sideband (LSB) and upper sideband (USB) with anti-phase are generated by a PM. The TBF is used to modify the relative amplitude of LSB and USB, resulting an asymmetric amplitude modulation signal. The PhC nanocavity is used as an optical filter. By adjusting the location of TBF to achieve different asymmetric amplitude modulation, a wide rejection tuning range from 11.8 to 62.1 dB is achieved experimentally. A wide central frequency tuning range from 12.9 to 32.3 GHz in high rejection ratio operating region of the proposed rejection ratio tunable MPF is also observed in the experiment.

## Results

### Concept and operation principle

**Figure 1:**
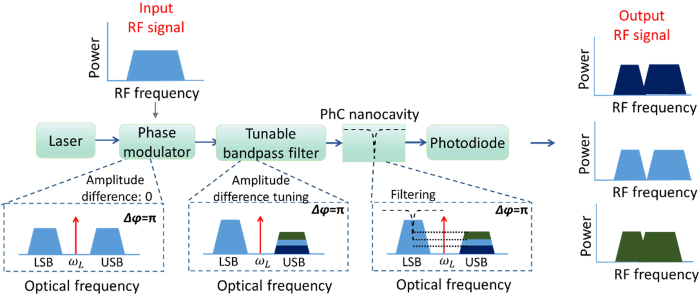
Schematic illustration of the proposed notch MPF with rejection ratio tunability.

Figure 1 summarizes the operation principle of the proposed rejection ratio tunable notch MPF based on a silicon PhC nanocavity. An optical carrier is modulated by a radio frequency (RF) signal with a conventional phase modulation. It is well known that the phase difference between the generated LSB and USB is *π*. If the modulated signal is applied directly to a photodiode (PD), due to the anti*-*phase relation between the LSB and USB, no RF signal could be obtained. Here the modulated signal is sent to a TBF first, and the USB signal is attenuated after the TBF. The output field is then applied to the PhC nanocavity. Note that the resonant frequency of the PhC nanocavity is aligned to the frequency of LSB signal, thus the LSB signal will be filtered by the PhC nanocavity. By tuning the location of the TBF, the relative amplitude of the LSB and USB can be tuned flexibly. Since the anti-phase condition of the LSB and USB, the amplitude difference of the LSB and USB will directly map to the rejection ratio of the MPF after detecting by the PD. Different amplitude difference between LSB and USB will lead to different rejection ratio of the obtained MPF. This is a photonic implementation of a rejection ratio tunable notch microwave filter, which exhibits rejection tunability from 0 dB to infinite rejection in principle.

### Theory

Providing a continuous wave (CW) is launched into a PM, the optical field at the output of the PM can be expressed as


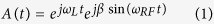


where *β* = *πV*_*RF*_*/V*_*π*_is the modulation index. *ω*_*L*_and *ω*_*RF*_ are the angular frequencies of the launched optical carrier and microwave signal applied on the PM, respectively. *V*_*RF*_ is the amplitude of the microwave signal. *V*_*π*_ is the half-wave voltage of the PM. Based on the Jacobi–Anger expansions, Eq. (1) can be expanded to be


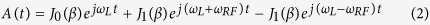


where *J*_*n*_ is the *n*th order Bessel function of the first kind. The the modulated signal is then sent to TBF and PhC nanocavity. At the output of PhC nanocavity, assuming the field transmission of the TBF and PhC nanocavity at *ω* is *T*_*TBF*_(*ω*) and *T*_*cavity*_(*ω*), respectively, the signal can be described as


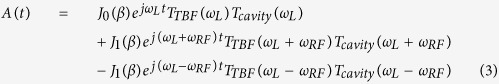


*T*_*cavity*_(*ω*) in the above equation is expressed by coupled mode theory^3^


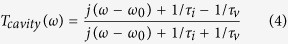


where *ω*_*o*_ is the resonant frequency of the PhC nanocavity. 1/*τ*_*i*_ is the photon lifetime reduction associated with the temporal coupling coefficients between PhC nanocavity and the input waveguide. 1/*τ*_*v*_ is the photon lifetime reduction in free space (vertical direction).

**Figure 2:**
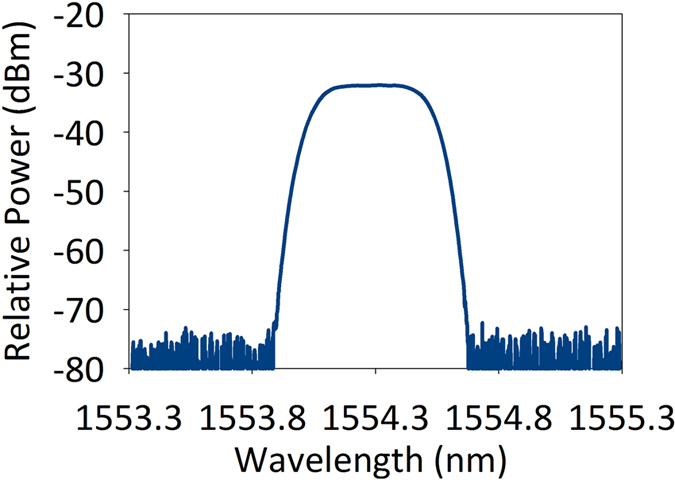
Measured transmission spectrum of the TBF.

**Figure 3:**
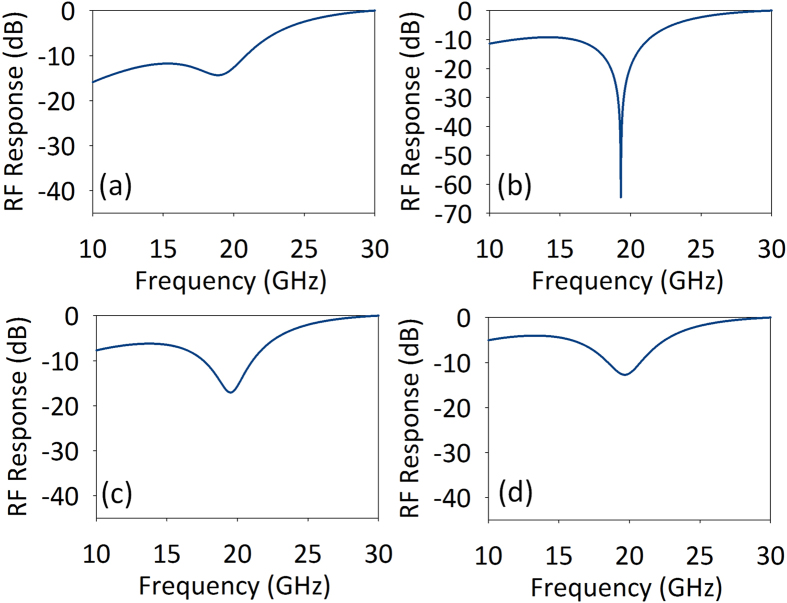
(**a–d**) Calculated MPF response when the central wavelength of the TBF is 1554.262 nm, 1554.292 nm, 1554.322 nm and 1554.352 nm, respectively.

We further calculate the RF response with different TBF location to show the realization of the proposed rejection ratio tunable MPF. In the simulation, the transmission spectrum of TBF is fitted from the measured transmission curve, which is shown in Fig. 2. We use a super-gaussian function 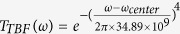 to fit the response of the TBF in the simulation. *ω*_*center*_ is the central angular frequency of the TBF. The parameters 1/*τ*_*i*_and 1/*τ*_*v*_ used to calculate PhC nanocavity field transmission are extracted from the measured PhC nanocavity transmission spectrum. The MPF response with different TBF locations is shown in Fig. 3(a–d). The carrier light wavelength is 1554.153 nm, and the central wavelength of TBF is increased from 1554.262 nm to 1554.352 nm with a step of 0.03 nm, in keeping with the experiment setup. It can be seen that when the central wavelength of TBF is 1554.292 nm, an very large rejection ratio MPF is obtained.

### Experiment

**Figure 4:**
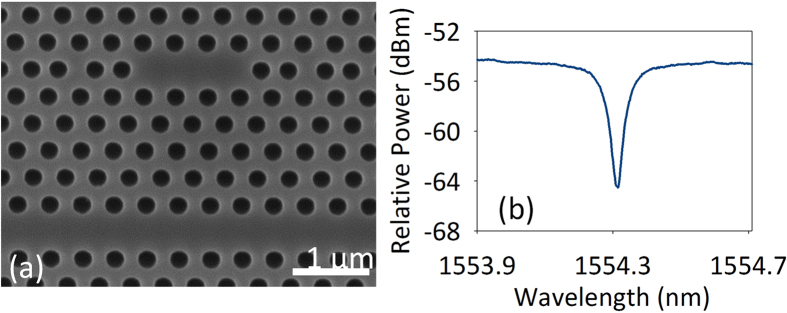
(**a**) Scanning electron microscope image of the PhC nanocavity and (**b**) its transmission spectrum.

The scanning electron microscope image of the fabricated PhC nanocavity is shown in Fig. 4(a). The PhC nanocavity consists of a PhC membrane with a line of three holes missing. The lattice constant is 420 nm, and the hole radius is 126 nm. Positions of the three holes adjacent to the cavity are optimized to obtain high Q factor. The three holes adjacent to the cavity are laterally shifted by 0.175*a*, 0.025*a*, 0.175*a*, respectively, where *a* is the lattice constant. Figure 4(b) shows the measured transmission spectrum of the fabricated PhC nanocavity. The resonant wavelength of the cavity is around 1554.313 nm.

**Figure 5:**
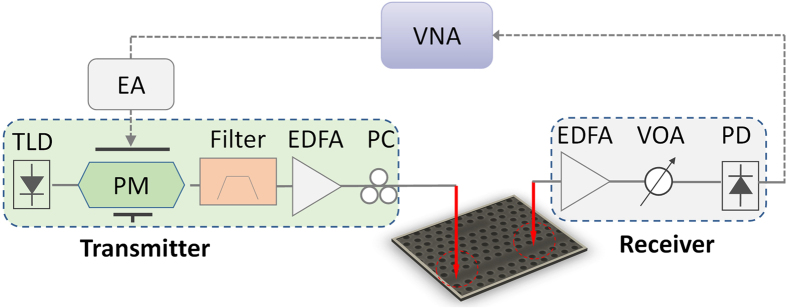
Experimental setup of bandpass MPF based on a silicon PhC nanocavity. Solid lines: optical path; dash lines: electrical path; TLD: tunable laser diode; PM: phase modulator; EDFA: erbium-doped fiber amplifier; VOA: variable optical attenuator; PC: polarization controller; PD: photodetector; EA: electrical amplifier; VNA: vector network analyzer.

**Figure 6:**
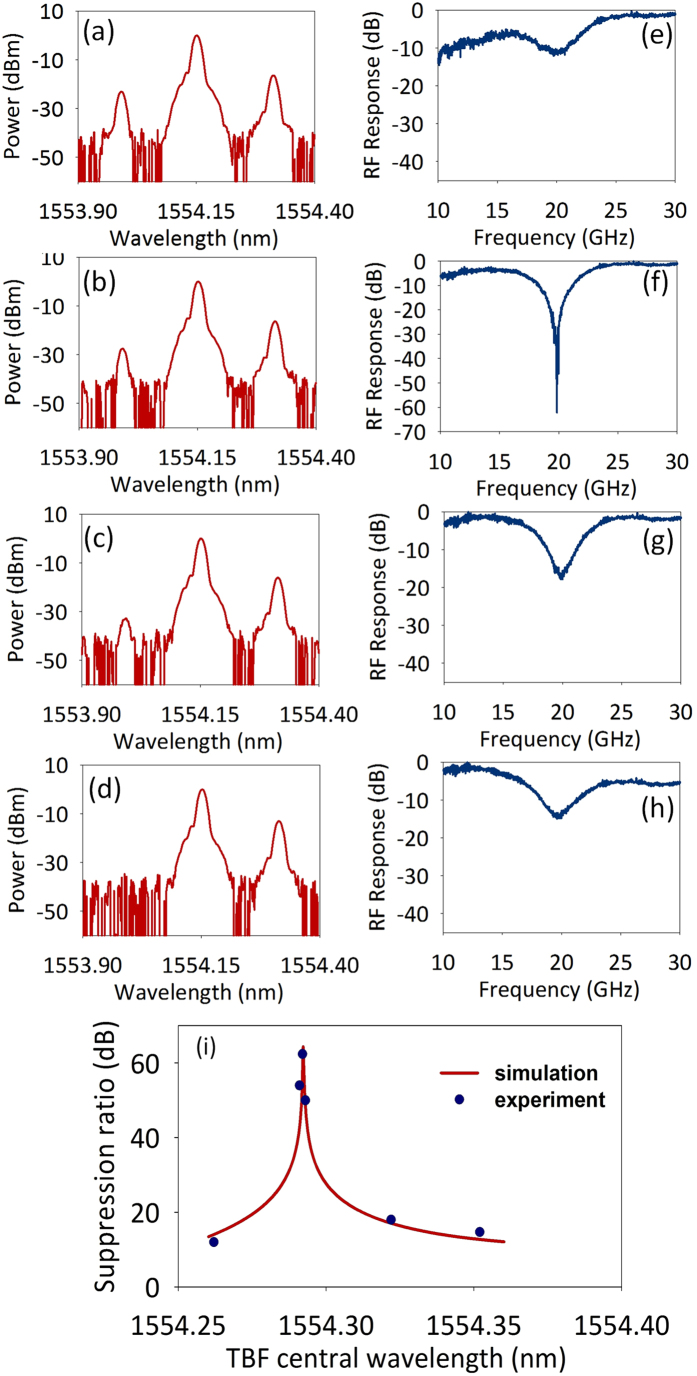
(**a–d**) Optical spectra after the TBF when the central wavelength of the TBF is 1554.262 nm, 1554.292 nm, 1554.322 nm and 1554.352 nm, respectively. (**e–h**) The corresponding MPF response. (**i**) Rejection ratio as a function of TBF central wavelength.

Figure 5 depicts the experimental setup. A tunable laser diode (TLD) emits a CW light. An electric amplifier (EA) is used to amplify the RF signal from vector network analyzer (VNA). The CW light is modulated by a PM to produce an optical double sideband signal. The TBF is used to modify the USB of the signal to obtain a modified asymmetric optical optical double sideband signal. The output field is then applied to the PhC nanocavity. After the device, the optical signal is converted to electric signal by a PD and analyzed by the VNA. The measured optical spectra after the TBF and the corresponding MPF responses are shown in Fig. 6. The carrier light wavelength is 1554.153 nm. Figure 6(a–d) depict the optical spectra when the central wavelength of the TBF is 1554.262 nm, 1554.292 nm, 1554.322 nm and 1554.352 nm, respectively. Figure 6(e–h) show the corresponding MPF responses. The experimental results agree well with the simulation. As shown in Fig. 6(a) and (e), when the USB signal is slightly modified by the TBF, the peak rejection of the obtained MPF is very small (~11.8 dB). When we shift the TBF, the power of USB signal decreases, and the peak rejection of the MPF increases. A maximum peak rejection of about 62.1 dB is observed when the central wavelength of the TBF is 1551.315 nm (Fig. 6(b) and (f)), owing to the frequency cancellation in high rejection ratio region. When we further increase the central wavelength of the TBF, the USB signal will be further attenuated, thus the peak rejection will decrease again. For example, as shown in Fig. 6(d) and (h), the peak rejection of the MPF decreases to 15 dB when the central wavelength of the TBF is 1551.375 nm. Figure 6(i) plots the rejection ratio as a function of the central wavelength of the TBF. The red curve shows the simulated data, while the experiemental results are marked by blue circles. A large rejection tuning range from 11.8 to 62.1 dB is observed, which is much larger than prevously reported rejection ratio tunable MPF^28^.

**Figure 7:**
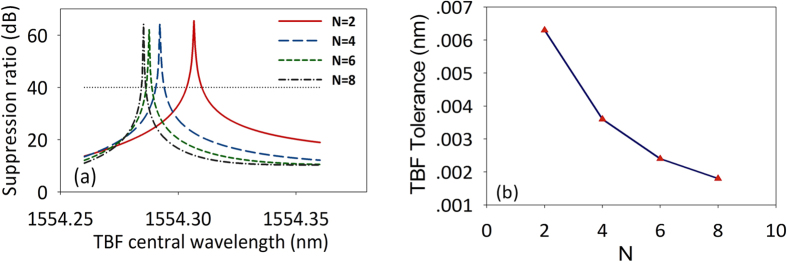
(**a**) Rejection ratio as functions of TBF central wavelength with different filter shape of TBF. (**b**) TBF tolerance of central wavelength with different filter shape of TBF.

To evaluate the effect of the filter shape of TBF, we study the performance of the rejection ratio tunable MPF with four types of super-gaussian filter shape. We calculate the MPF tunability response when the transmission of the TBF is 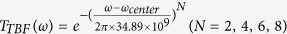. Figure 7(a) shows the rejection ratio of the MPF as a function of TBF central wavelength with different filter shapes of TBF. To show the operation stability in high rejection ratio region with different TBF filter shapes, we define the TBF central wavelength tolerance as operation span of TBF central wavelength when the rejection ratio is greater than 40 dB. Figure 7(b) plots the TBF tolerance of central wavelength with different filter shape of TBF. It can be seen that higher order super-gaussian function leads to worse stability of the system.

**Figure 8:**
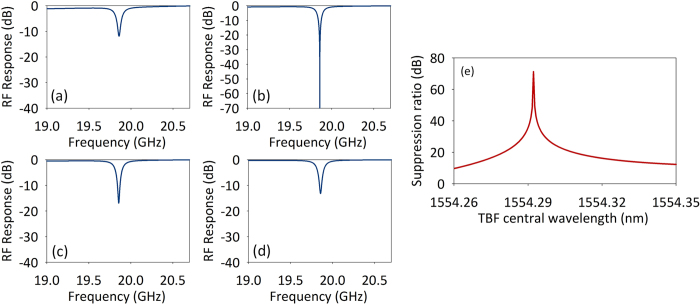
Calculated (**a–d**) MPF response when the central wavelength of the TBF is 1554.262 nm, 1554.292 nm, 1554.322 nm and 1554.352 nm, respectively and (**e**) rejection ratio versus TBF central wavelength when the linewidth of the PhC nanocavity is narrow.

The proposed rejection ratio tunable MPF could also work well when the linewidth of the PhC nanocavity is very narrow. Figure 8(a–d) show the calculated MPF response when the central wavelength of the TBF is 1554.262 nm, 1554.292 nm, 1554.322 nm and 1554.352 nm, respectively, where the bandwidth of the MPF is about 60 MHz. The rejection ratio as a function of TBF central wavelength is plotted in Fig. 8(e).

**Figure 9:**
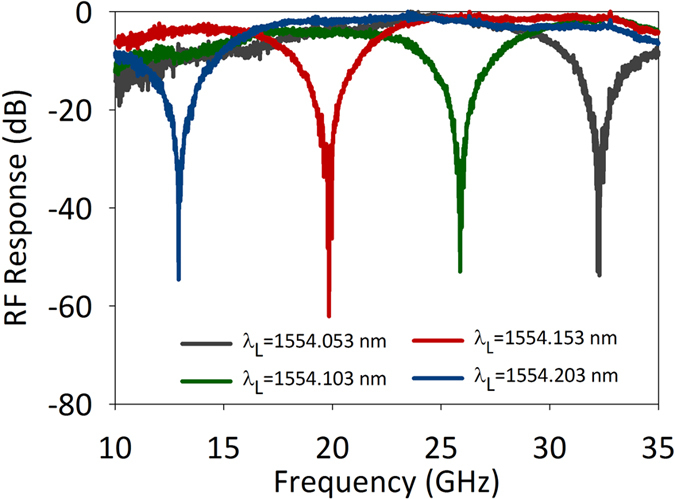
Measured central frequency tunability of the proposed MPF.

By changing the carrier light wavelength, the operating frequency of the rejection ratio tunable MPF can also be tuned. To comprehensively assess the tunable operation of the MPF, we measure the operating frequency tunability of the MPF when working in the high rejection ratio region, as shown in Fig. 9. When the wavelength of the carrier light is changed from 1554.053 to 1554.203 nm, the central frequency of the MPF is tuned from 32.3 to 12.9 GHz, maintaining a high rejection ratio. The obtained central frequency tuning range is 19.4 GHz, which is much large than the previous reported rejection ratio tunable MPF^28^. The wide tuning range is benefit from the very wide free spectral range of the PhC nanocavity. It should be noted that the central frequency of the proposed MPF can be continuous tuned from DC to a much higher frequency (several hundreds of GHz) in principle. The measured results here is limited by the bandwidth of our test equipment.

## Method

### Devices fabrication

The PhC nanocavity is fabricated on an SOI wafer. Electron beam lithography and inductively coupled plasma etching are used to define patterns on an SOI wafer (220-nm-thick silicon on 3000-nm-thick silica). The cavity is processed by diluted hydrofluoric acid solution to strengthen optical confinement in the normal direction and increase the symmetry of the structure. The whole fabrication process is done using CMOS compatible processes.

## Additional Information

**How to cite this article**: Long, Y. *et al*. Photonic crystal nanocavity assisted rejection ratio tunable notch microwave photonic filter. *Sci. Rep.* **7**, 40223; doi: 10.1038/srep40223 (2017).

**Publisher's note:** Springer Nature remains neutral with regard to jurisdictional claims in published maps and institutional affiliations.
